# The beneficial fungus *Piriformospora indica* protects Arabidopsis from *Verticillium dahliae* infection by downregulation plant defense responses

**DOI:** 10.1186/s12870-014-0268-5

**Published:** 2014-10-09

**Authors:** Chao Sun, Yongqi Shao, Khabat Vahabi, Jing Lu, Samik Bhattacharya, Sheqin Dong, Kai-Wun Yeh, Irena Sherameti, Binggan Lou, Ian T Baldwin, Ralf Oelmüller

**Affiliations:** Institute of Plant Physiology, Friedrich-Schiller-University Jena, Dornburger Str. 159, 07743 Jena, Germany; Max Planck Institute for Chemical Ecology, Hans-Knöll-Str. 8, D-07745 Jena, Germany; College of Life Sciences, Yangtze University, Jingzhou, China; Institute of Plant Biology, National Taiwan University, Taipei, Taiwan; Institute of Biotechnology, Zhejiang University, Hangzhou, 310058 China; Institute of Insect Sciences, Zhejiang University, Hangzhou, 310058 China

**Keywords:** Calcium, Defense, Ethylene, Jasmonic acid, *Piriformospora indica*, Salicylic acid, *Verticillium dahliae*

## Abstract

**Background:**

*Verticillium dahliae* (*Vd*) is a soil-borne vascular pathogen which causes severe wilt symptoms in a wide range of plants. The microsclerotia produced by the pathogen survive in soil for more than 15 years.

**Results:**

Here we demonstrate that an exudate preparation induces cytoplasmic calcium elevation in Arabidopsis roots, and the disease development requires the ethylene-activated transcription factor EIN3. Furthermore, the beneficial endophytic fungus *Piriformospora indica* (*Pi*) significantly reduced *Vd*-mediated disease development in Arabidopsis. *Pi* inhibited the growth of *Vd* in a dual culture on PDA agar plates and pretreatment of Arabidopsis roots with *Pi* protected plants from *Vd* infection. The *Pi*-pretreated plants grew better after *Vd* infection and the production of *Vd* microsclerotia was dramatically reduced, all without activating stress hormones and defense genes in the host.

**Conclusions:**

We conclude that *Pi* is an efficient biocontrol agent that protects Arabidopsis from *Vd* infection. Our data demonstrate that *Vd* growth is restricted in the presence of *Pi* and the additional signals from *Pi* must participate in the regulation of the immune response against *Vd*.

**Electronic supplementary material:**

The online version of this article (doi:10.1186/s12870-014-0268-5) contains supplementary material, which is available to authorized users.

## Background

Verticillium species are wide-spread soil-borne fungi which cause vascular diseases in many plant species and are responsible for devastating diseases for plants that can thwart agricultural production. The vascular wilt fungus *Verticillium dahliae* (*Vd*), for instance, infects more than 200 plant species, among them agriculturally and horticulturally important crops and ornamental plants [1-3]. It is estimated that *Vd* infections are responsible for several billions of dollars of annual crop losses worldwide. *Vd* has a broad host range and infects plants from temperate to subtropical climates [1]. Because of their complex life style of the Verticillium species, their control by classical pesticides or fungicides is difficult; therefore, the isolation of Verticillium-resistant cultivars is an important task for the breeders (cf. [4,5]).

Genetic resistance against Verticillium wilt diseases has been reported for several plant species [1,2]. The *Ve* gene provides resistance against race 1 isolates of *Vd* in tomato [6,7] and the tomato gene is also functional after expression in Arabidopsis [8]. Many studies have used Arabidopsis for the isolation of *Vd*-resistant germplasm [9,10] or the identification of novel resistance traits following mutagenesis [2,10-14]. Furthermore, quite recently, a large number of proteins and metabolites from different organisms as well as phytohormones have been described to be involved in establishing partial resistance against Verticillium wilt [15-22].

Like other Verticillium species, *Vd* can overwinter as mycelium in host plants or soil. The fungus can also form seed-like structures called microsclerotia, long-lived survival structures of clusters of melanized cells with thick walls, which survive in the soil without a host plant or in association with plant material for up to 20 years [23,24]. The microsclerotia germinate in response to stimuli from root exudates [25]. The hyphae penetrate and grow inter- and intracellularly through the root cortex toward the central cylinder of the root [26,27]. They enter the xylem cells of the root, from where they colonize the xylem of the hypocotyl and leaves. Ultimately, the water transport is disrupted which results in the wilt phenotype [1-3]. Verticillium species are considered as hemibiotroph: a biotrophic phase within root xylem without a visible disease phenotype is followed by a necrotrophic phase in the aerial parts of the plant.

The spread of the pathogen occurs primarily by root infections from the soil. Therefore rhizosphere bacterial strains such as *Pseudomonas putida* B E2, *Pseudomonas chlororaphis* K15 or *Serratia plymuthica* R12 [28] or bacterial isolates [29] have been shown to function as efficient biocontrol agents against *Vd* spread. The microbial bioagents induce antibiosis, parasitism, competition and secretion of enzymes such as glucose oxidase, chitinase and glucanase which results in the induction of disease resistance in the hosts [12,30].

To our knowledge, there is no report on endophytic fungi which can be used as biocontrol agent against *Vd* in Arabidopsis. *Piriformospora indica* (*Pi*), a cultivable basidiomycete of Sebacinales, colonizes the roots of many plant species including Arabidopsis [31,32]. Like other members of Sebacinales, *Pi* is found worldwide in association with roots [33] and stimulates growth, biomass and seed production of the hosts [31,34-36]. The fungus promotes nitrate and phosphate uptake and metabolism [35,37]. *Pi* also confers resistance against abiotic [38,39] and biotic stress [40].

Here, we demonstrate that *Pi* is an efficient biocontrol agent that protects Arabidopsis from *Vd* infection *in vitro* and *in vivo* by inhibiting growth of *Vd* in roots. Furthermore, we give evidence that a *Vd*-exudate compound induces cytoplasmic Ca^2+^ ([Ca^2+^]_cyt_) elevation and the *Vd*-disease development is dependent on the ethylene-activated transcription factor EIN3.

## Results

### *Pi* inhibits growth of *Vd* on PDA agar plates

**Figure 1:**
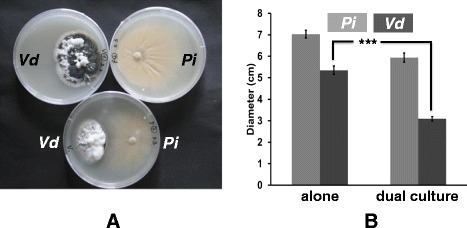
***Pi*** **inhibits growth of** ***Vd*** **on agar plates. (A)** Typical plates from 3 independent experiments are shown. **(B)** Quantification of the colony. The diameter of the *Pi* and *Vd* mycelia on the agar plate is given in cm. Bars represent SDs. Asterisks indicate significant differences, as determined by ANOVA (*** P ≤ 0.001).

*Pi* and *Vd* were co-cultivated as described in Methods on a PDA agar plate for 3 weeks. Figure 1(A and B) demonstrates that *Pi* strongly inhibits growth of *Vd* hyphae. The *Vd* colony in the dual culture is significantly smaller than the *Vd* colony growing without *Pi*. Furthermore, the number of microsclerotia produced by *Vd* in the dual culture is less than the number of microsclerotia produced by *Vd* growing alone. No obvious inhibition zone can be detected. In contrast, growth of *Pi* is barely affected by the presence of *Vd*. This prompted us to test the role of *Pi* in protecting Arabidopsis plants against *Vd* infection.

### Arabidopsis seedlings pretreated with *Pi* are protected against *Vd* infection

**Figure 2:**
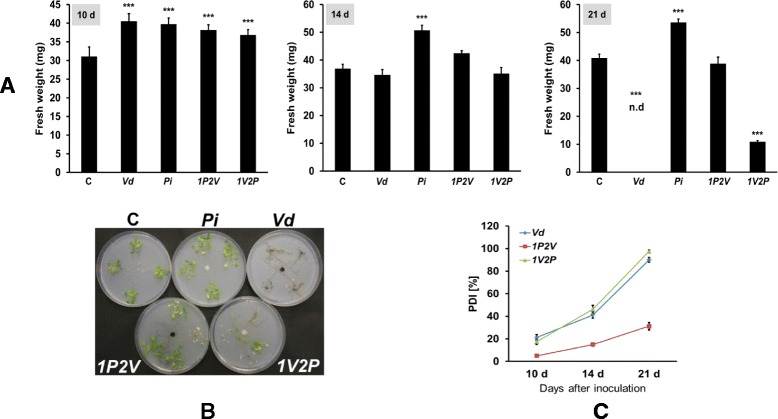
***Pi*** **protects Arabidopsis seedlings from** ***Vd*** **infection. (A)** Fresh weights of seedlings after 10, 14 and 21 days of co-cultivation or mock-treatments on Petri dishes. The seedlings were exposed to either *Pi* or *Vd* alone or in combination as described in the Methods and Additional file 1: Figure S1. C: seedlings treated without fungi; *Vd*: seedlings treated with *Vd*; *Pi*: seedlings treated with *Pi*; *1P2V*: seedlings first treated with *Pi* for 4 days followed by *Vd*; *1V2P*: *vice-versa* as *1P2V*. n.d: no detectable (seedlings were dead, no fresh weight could be determined). The data are based on 3 independent experiments with 16 seedlings each. Bars represent SDs. Asterisks indicate significant differences, as determined by ANOVA (* P ≤ 0.05; ** P ≤ 0.01; *** P ≤ 0.001). **(B)** The phenotype of typical seedlings on 21^st^ day. **(C)** PDI for seedlings exposed to *Vd*. For treatments, cf. Methods and Additional file 1: Figure S1. The data are based on 3 independent experiments with 16 seedlings each. Bars represent SDs.

To investigate whether *Pi* can protect Arabidopsis for *Vd* infection, we exposed the seedlings first to *Pi* prior to *Vd* infection. Seedlings not exposed to any of the two fungi or to one of the two fungi alone served as controls (cf. Methods). The performance of the seedlings was measured after 10, 14 and 21 days, by visible inspection and measuring the fresh weights. After 10 days of co-cultivation, seedlings treated with *Vd* or *Pi* alone showed ~30% increase in the biomass compared to the untreated control seedlings. A comparable increase in the biomass was observed when the seedlings were first exposed to *Pi* and then to *Vd* or *vice versa* (Figure 2A). This slight increase in the biomass suggests that both fungi initially form a beneficial interaction with the seedlings, and is consistent with the idea that this phase represents a biotrophic interaction of *Vd* with Arabidopsis roots. On the 14^th^ day, seedlings infected by *Vd* alone or first with *Vd* followed by *Pi* (*1V2P*) showed obviously the disease symptoms. The leaves of these seedlings became paler and the roots browner compared to the seedlings exposed to *Pi* or *1P2V* treatments, although no significant differences in the biomass were observed for the different fungal treatments, except for *Pi* treatment (Figure 2A). In contrast, on the 21^st^ day, seedlings exposed to *Vd* alone or exposed to *Vd* prior to exposure to *Pi* (*1V2P*) were severely damaged. Their fresh weights were reduced or no longer measurable. *Pi* treatment alone resulted in a ~30% increase in the fresh weight (Figure 2A). Interestingly, seedlings which were pretreated with *Pi* and then exposed to *Vd* (*1P2V*) had the same fresh weights as untreated control seedlings, although the visible inspection showed some photo-bleaching (Figure 2B). This clearly demonstrates that *Pi* protects Arabidopsis seedlings against *Vd*-induced wilt. Therefore, this experimental set-up was used to study the protective function of *Pi* in greater details.

The results were confirmed by calculating the Percentage Disease Index (PDI) for those seedlings treated with *Vd*. After 10 days of co-cultivation, the PDI for *Vd* and *1V2P* seedlings was ~20%, and after 14 days 40-50%. After 21 days, the PDI was almost 100%. In contrast, seedlings pretreated with *Pi* prior to exposure to *Vd* (*1P2V*) showed a slow increase in the PDI, which reached ~30% after 21 days (Figure 2C).

**Figure 3:**
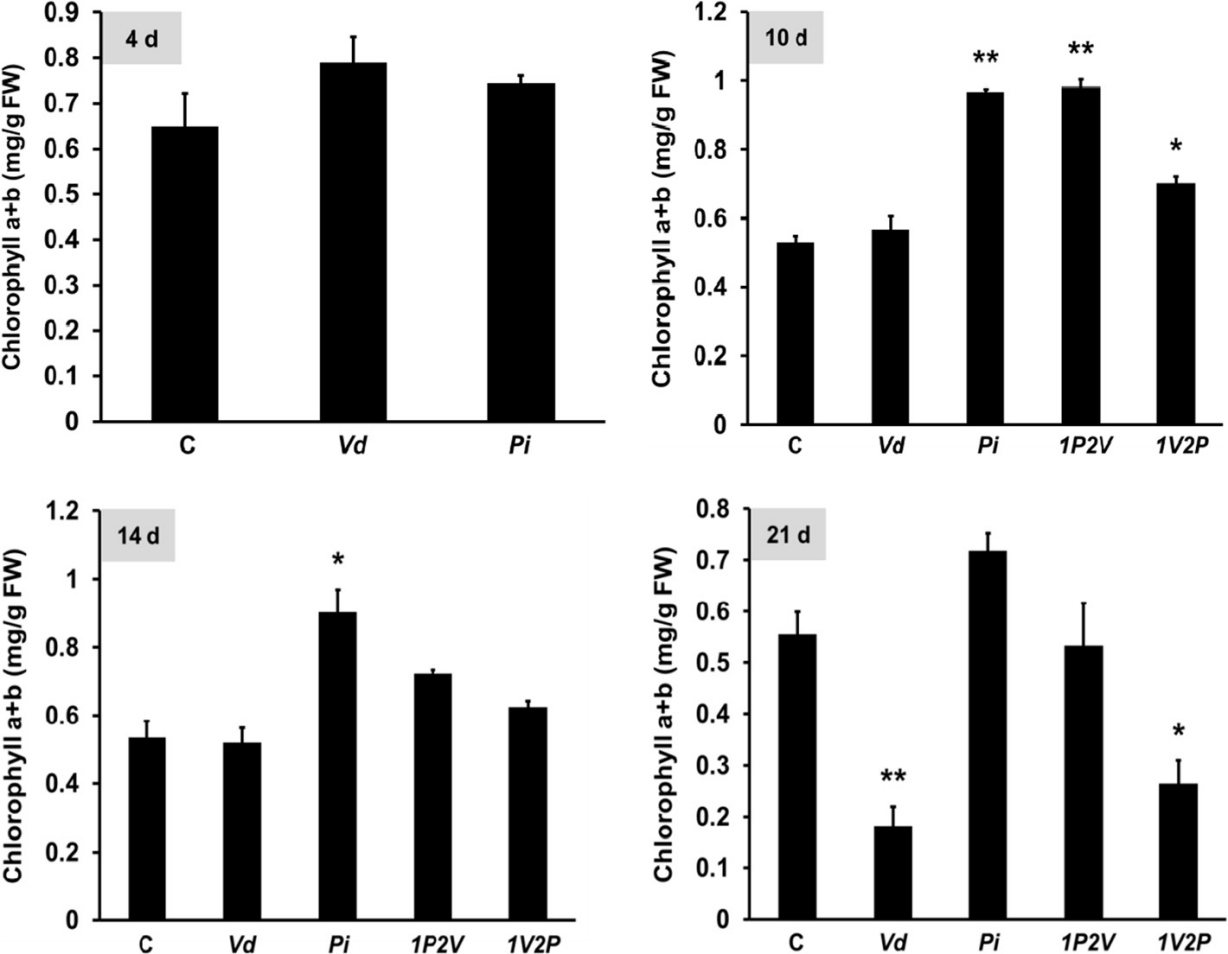
**Total chlorophyll content (mg/g fresh weight) in shoots.** The data were obtained 4, 10, 14 and 21 days after the fungal treatments (cf. Methods, Additional file 1: Figure S1 and legend to Figure 2A). The data are based on 3 independent experiments with 16 seedlings each. Bars represent SDs. Asterisks indicate significant differences to the untreated control, as determined by Student’s t-test (* P ≤ 0.05; ** P ≤ 0.01; *** P ≤ 0.001).

Furthermore, the amount of total chlorophyll (Chl) is a sensitive marker for the fitness of a plant. On the 4^th^ day, the shoots of *Vd-* and *Pi-* treated plants contained slightly higher Chl levels than control seedlings (Figure 3). On the 10^th^ day, the Chl content of *Vd-* treated seedlings is comparable to that of control seedlings not exposed to the pathogen. Furthermore, while *1P2V* seedlings had the same amount of Chl as *Pi* seedlings, the Chl content in *1V2P* seedlings was significantly reduced (Figure 3). Comparable results were obtained for the 14^th^ day, except that the Chl content for *1P2V* seedlings was reduced compared to *Pi* seedlings (Figure 3). On the 21^st^ day, *Pi* seedlings had the highest Chl content, *1P2V* seedlings had the same amount of Chl as control seedlings not exposed to a fungus, while the Chl levels in the *Vd* and *1V2P* plants were strongly decreased (Figure 3). This confirms the protective function of *Pi* against *Vd* infection in Arabidopsis leaves.

**Figure 4:**
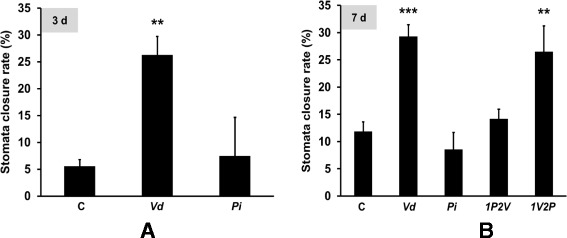
**Stomata closure rate in leaves after 3 (A) and 7 (B) days.** The data are based on 3 independent experiments with 16 seedlings each. Bars represent SDs. Asterisks indicate significant differences to the untreated control, as determined by Student’s t-test (* P ≤ 0.05; ** P ≤ 0.01; *** P ≤ 0.001).

Pathogenesis and application of pathogen-associated molecular patterns induce stomata closure [41]. In control plants not exposed to any fungus, between 5 and 12% of the stomata were closed. Three days after exposure of the roots to *Vd*, ~25% of the stomata were closed (Figure 4A), and this increased to ~30% until the 7^th^ day. The *1V2P* treatment showed ~25% stomata closure at the 7^th^ day, and this value is comparable to that for seedlings treated with *Vd* alone. In contrast, exposure of the roots to *Pi* or first to *Pi* followed by *Vd* did not result in stomata closure and these values are comparable to those of the untreated controls (Figure 4B). This indicates that *Pi* prevents *Vd*-induced stomata closure. These results demonstrate that stomatal closure correlates nicely with the amount of total chlorophyll.

### *Pi* represses *Vd*-induced genes in shoots

**Figure 5:**
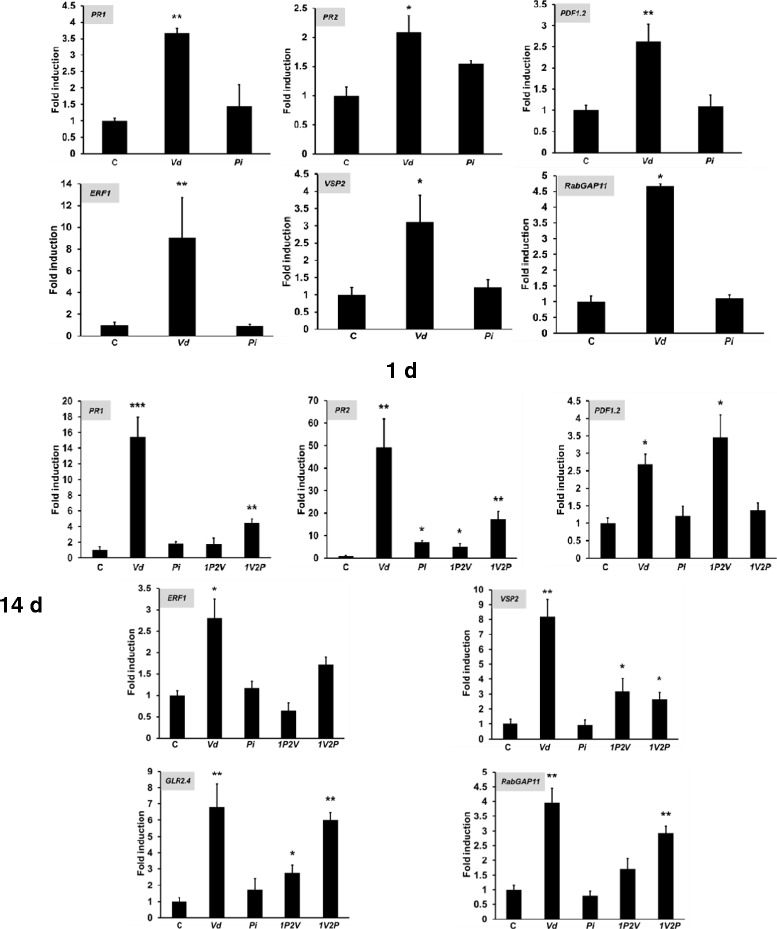
**Induction of defense genes in the shoots of Arabidopsis seedlings 1 and 14 days after the fungal treatments, relative to the untreated control.** The data represents fold induction (mRNA level _+fungal treatments_/mRNA level _-fungal treatments_; fold of control is set as 1.0). For experimental details, cf. Methods, Additional file 1: Figure S1 and legend to Figure 2A. The data are based on 5 independent experiments with 16 seedlings each. Bars represent SDs. Asterisks indicate significant differences, as determined by Student’s t-test (* P ≤ 0.05; **P ≤ 0.01; *** P ≤ 0.001).

*Vd* induces defense gene expression in shoots. After 1 d, the mRNA levels for *PR1* and *PR2* representing SA-inducible genes and *PDF1.2* for the JA/ET pathway, *ERF1* and *VSP2* for ET pathway were upregulated in the leaves of *Vd*-exposed seedlings. Except for *PR2*, none of the other genes responded to *Pi* exposure (Figure 5). After 14 d, *Vd-*exposed seedlings showed an even stronger upregulation of the defense genes in the leaves (Figure 5). Pretreatment of the seedlings with *Pi* prior to *Vd* infection resulted in the repression of defense gene expression compared to seedlings which were not pretreated with *Pi*. This provides additional evidence for the protective function of *Pi* against *Vd* infection. Furthermore, plant glutamate receptor-like (*GLR*) genes, *GLR2.4*, *GLR2.5* and *GLR3.3* code for putative Ca^2+^ transporters and are involved in defense responses [42-44]. We observed that *GLR2.4* (but not *GLR2.5* and *GLR3.3*) was upregulated in the leaves of *Vd*-exposed seedlings and repressed in the leaves of seedlings which were pretreated with *Pi* prior to *Vd* exposure (Figure 5 and Additional file 1: Figure S2). *RabGAP22* is required for defense to *V. longisporum* and contributes to stomata immunity [22]. For *Vd*, *RabGAP11* is upregulated after exposure to *Vd* and significantly repressed in seedlings which were pretreated with *Pi* (Figure 5).

### *Pi* strongly represses *Vd*-induced phytohormone accumulation in shoots

**Figure 6:**
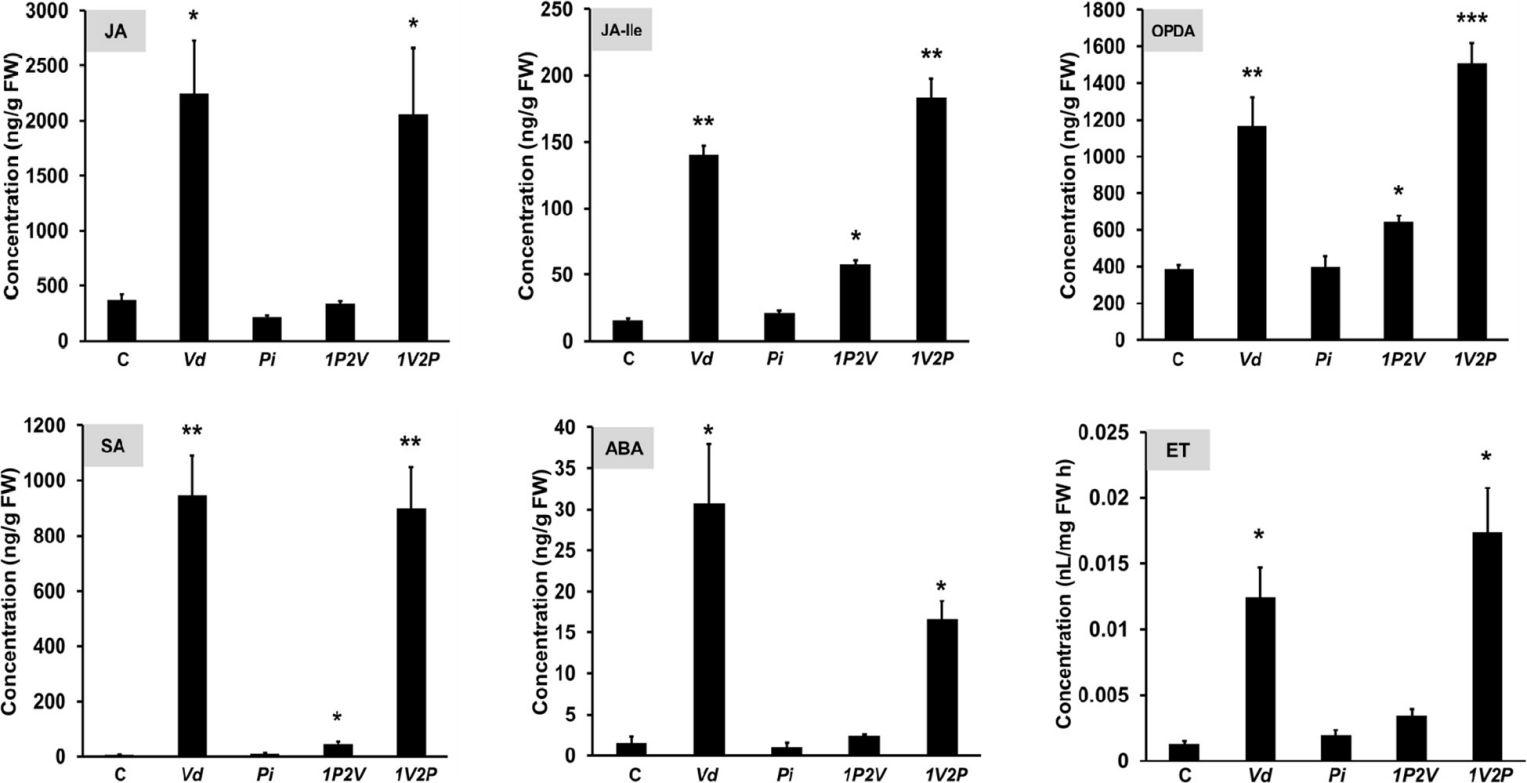
**Phytohormone levels in the shoots 21 days after the different fungal treatments.** For experimental details, cf. Methods, Additional file 1: Figure S1, and legend to Figure 2A. The data are based on 3 independent experiments with 12 seedlings each. Bars represent SDs. Asterisks indicate significant differences, as determined by Student’s t-test (* P ≤ 0.05; ** P ≤ 0.01; *** P ≤ 0.001).

The phytohormones JA, JA-Ile, OPDA, SA, ABA and ET are crucial for the activation of defense responses. Figure 6 demonstrates that these phytohormones accumulated after *Vd* infection in the shoots of Arabidopsis seedlings. The phytohormone levels were also high in the *1V2P* samples, while in all other cases [Control (C), *Pi*, *1P2V*], they showed significantly lower levels. Thus, *Vd*-induced phytohormone accumulation is repressed if the roots are colonized by *Pi* prior to their exposure to *Vd*. Interestingly, application of *Pi* to roots which were already exposed to *Vd* did not repress the accumulation of the phytohormones in the shoots.

### *Pi* inhibits *Vd* propagation and microsclerotia formation

**Figure 7:**
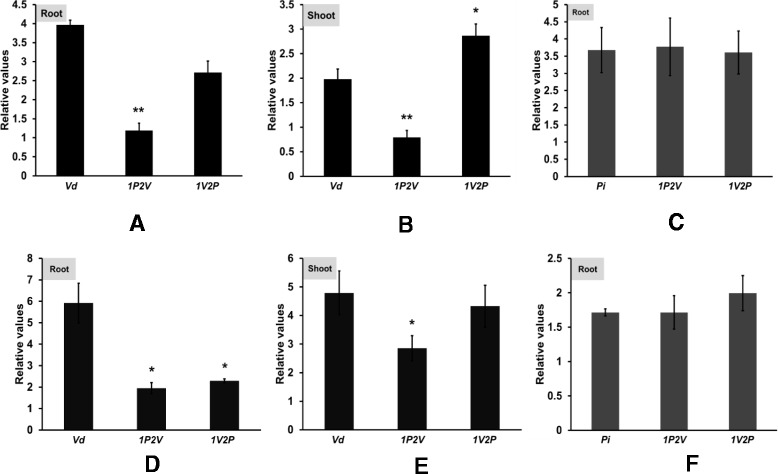
**The amount of fungal DNA in the roots and shoots of Arabidopsis seedlings exposed to the 5 treatments (cf. legend to Figure** **2** **A).** For experimental details, cf. Methods and Additional file 1: Figure S1. The measurements were performed for the 14^th^ **(A, B, C)** and 21st **(D, E, F)** day. The data are based on 3 independent experiments with 12 seedlings each. Bars represent SDs. Asterisks indicate significant differences compared to *Vd* **(A, B, D, E)** or to *Pi* **(C and** **F)**, as determined by Student’s t-test (* P ≤ 0.05; ** P ≤ 0.01; *** P ≤ 0.001).

**Figure 8:**
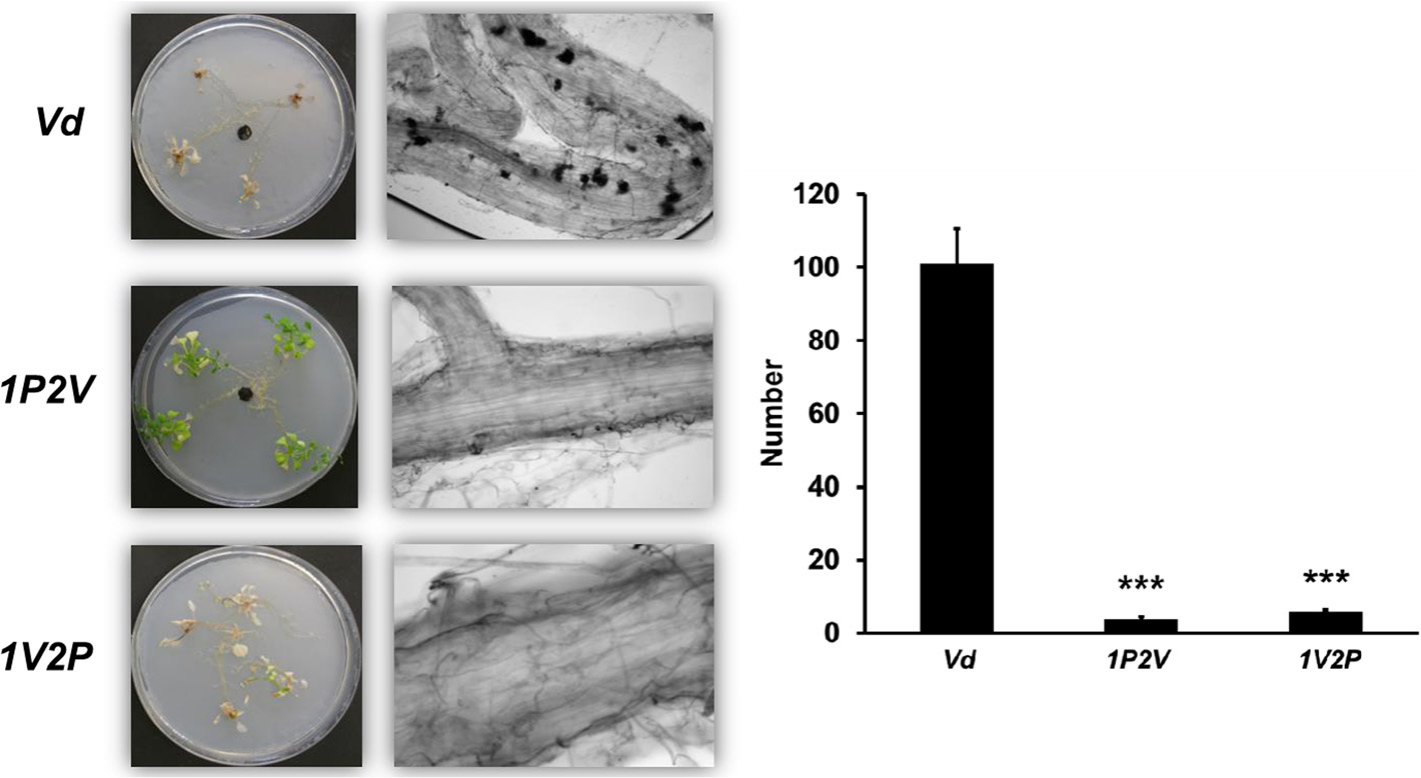
***Pi*** **inhibits the formation of** ***Vd*** **micosclerotia in roots, irrespective of whether the roots were first exposed to** ***Pi*** **(** ***1P2V*** **) or first to** ***Vd*** **(** ***1V2P*** **).** The analysis was performed 21 days after infection. Left: microscopy of root sections with microslerotia (black spots). Right: Quantification of the number of microsclerotia. The data are based on 3 independent experiments with 12 seedlings each. Bars represent SDs. Asterisks indicate significant differences to *Vd*, as determined by Student’s t-test (* P ≤ 0.05; ** P ≤ 0.01; *** P ≤ 0.001).

Quantification of the amount of *Vd* DNA demonstrated that *Vd* and *1V2P* seedlings contain twice as much pathogen DNA than *1P2V* seedlings in both roots (Figure 7A and D) and shoots (Figure 7B and E). Interestingly, the amount of *Pi* DNA in the roots is identical in all *Pi*-treated samples and not affected by a pretreatment with *Vd* (Figure 7C and F). Furthermore, microscopic analysis demonstrated that the number of microsclerotia was strongly reduced in root tissue pretreated with *Pi* (Figure 8). This demonstrates that *Pi* inhibits *Vd* propagation and microsclerotia formation in the roots, while *Vd* does not affect the propagation of *Pi* in Arabidopsis roots.

### Long-term experiments confirmed the results obtained for seedlings

**Figure 9:**
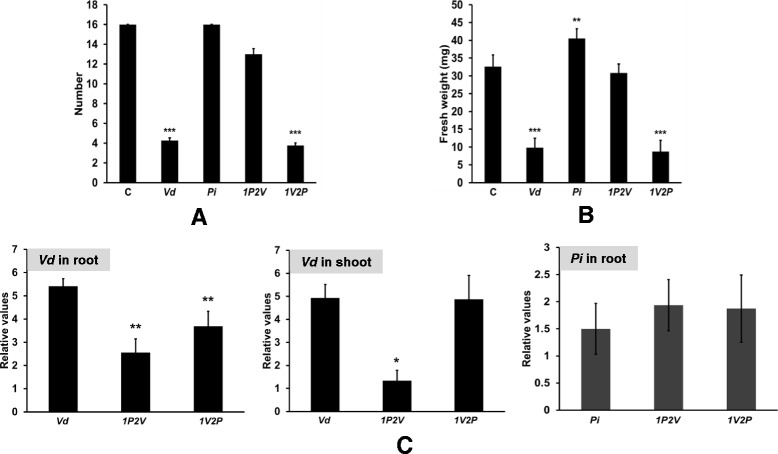
**Confirmation of the results for adult plants, grown in sterile vermiculite.** After exposure of the seedlings to the 5 treatments in Petri dishes for 10 days (cf. legend to Figure 2A), they were transferred to Magenta boxes with sterile vermiculite for 14 days. **(A)** Number of survived plants. **(B)** Fresh weight of plants. **(C)** Fungal DNA content in roots and shoots. The data are based on 3 independent experiments with 16 seedlings each. Bars represent SDs. Asterisks indicate significant differences to *Vd*, as determined by Student’s t-test (* P ≤ 0.05; ** P ≤ 0.01; *** P ≤ 0.001).

In order to study long term interaction, the seedlings were grown according to the 5 regimes on Petri dishes for 10 days before transferred to sterile vermiculite for additional 14 days. All (C) seedlings and those exposed to *Pi* (*Pi*) were alive. Exposure of *Pi*-pretreated plants to *Vd* resulted in ~20% loss of the plants. However 80% of the plants, which were either exposed to *Vd* alone or first to *Vd* followed by *Pi*, died (Figure 9A). Furthermore, we measured the fresh weights of the seedlings which survived the treatments. Plants exposed to *Pi* alone showed a ~30% increase in the fresh weight. The fresh weights of *1P2V* plants were comparable to those not exposed to any fungus. *Vd*- and *1V2P*-treated seedlings showed significantly decreased fresh weights compared to all other treatments (Figure 9B). Finally, the *Vd* DNA amount in both shoots and roots was lower in *1P2V*-treated plants compared to those treated with *Vd* alone or first with *Vd* followed by *Pi* (*1V2P*) (Figure 9C). Comparable to the results obtained with seedlings in Petri dishes (Figure 7), the *Pi* DNA content was the same in all *Pi*-treated roots (Figure 9C). This confirms that *Pi* inhibits *Vd* growth, but not *vice versa*.

### EIN3 is required for full susceptibility of Arabidopsis to *Vd*

**Figure 10:**
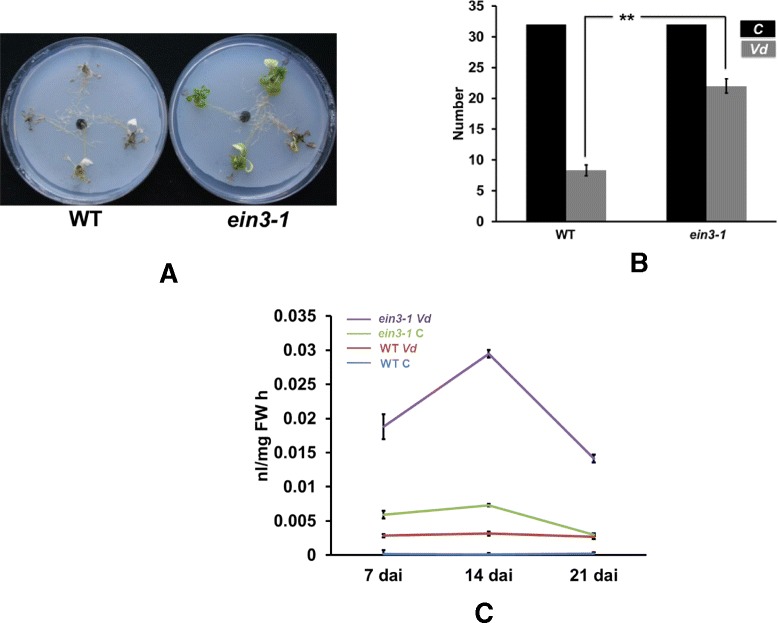
**EIN3 is required for full susceptibility of Arabidopsis to** ***Vd*** **. (A)** The representative picture (3 independent experiments with 32 plants each) was taken after 21 days inoculation with *Vd*. **(B)** Number of survived seedlings. **(C)** Ethylene levels in WT and *ein3* seedlings after exposure to *Vd*. Bars represent SDs. Asterisks indicate significant differences, as determined by Student’s t-test (* P ≤ 0.05; ** P ≤ 0.01; *** P ≤ 0.001).

The strong upregulation of the phytohormone levels in the leaves of seedlings grown in the presence of *Vd* was further investigated for ET. Pantelides et al. [11] have shown that ET perception *via* ETR1 is required for *Vd* infection in Arabidopsis. We observed a strong requirement of EIN3 for *Vd*-induced disease development in Arabidopsis leaves. *ein3* seedlings which were exposed to *Vd* alone or were first treated with *Vd* before application of *Pi* perform better than wild-type seedlings (Figure 10A, B and Additional file 1: Figure S3). Interestingly, the ET level in *ein3* seedlings is much higher than in wild-type seedlings, even in the absence of *Vd*. Exposure of the seedlings to *Vd* stimulate ET accumulation even further (Figure 10C and Additional file 1: Figure S4). This suggests that *ein3* seedlings try to compensate the lack of EIN3-induced genes by further stimulating ET biosynthesis, in particular after *Vd* infection. Taken together, these data demonstrate that EIN3-induced genes are required for pathogenicity of *Vd*.

### *Vd* induces [Ca^2+^]_cyt_ elevation in WT roots, but not in roots of a Ca^2+^ response mutant

**Figure 11:**
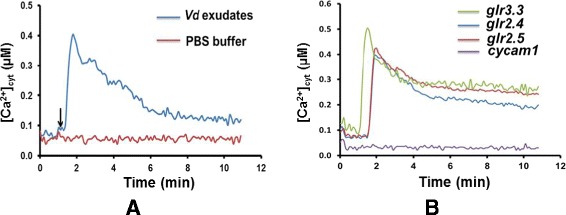
***Vd*** **exudate preparation induces [Ca** ^**2+**^ **]**_**cyt**_ **elevation in** ***A. thaliana*** **seedlings expressing cytosolic aequorin. (A)** Roots of 21-day old pMAQ2 in Col-0 seedlings were dissected and incubated overnight in 7.5 μM coelenterezine. The roots were challenged with 50 μl of the *Vd* preparations. [Ca^2+^]_cyt_ level was calculated from the relative light unit (RLU) at 5 s integration time for 10 min. The arrow indicates the time (60 s) of addition of the stimuli/PBS buffer. For all experiments, 10 mM phosphate buffer (PBS, pH 7.0) was used as control and gave background readings. All curves and values represent average of five independent experiments with eight replications in each experiment. **(B)** *Vd* exudate preparation does not induce [Ca^2+^]_cyt_ elevation in the *cycam1* mutant, but induces [Ca^2+^]_cyt_ elevation in pMAQ2 lines in the *glr2.4*, *glr2.5* and *glr3.3* background.

Pathogen-associated molecular pattern-triggered immunity is often initiated by [Ca^2+^]_cyt_ elevation, which can be induced by exudated compounds from pathogenic fungi [cf. [45] and ref. therein]. Since the putative plasma membrane-localized Ca^2+^-transporter gene *GLR2.4* was upregulated by *Vd*, we tested whether exudated compounds from *Vd* can induce [Ca^2+^]_cyt_ elevation in roots. An exudate preparation from the mycelium was applied to the roots of transgenic pMAQ2 Arabidopsis lines expressing the Ca^2+-^sensor apoaequorin. Under resting conditions, 21 d-old pMAQ2 lines gave [Ca^2+^]_cyt_ values of 70 ± 0.6 nM (*n* = 16). A rapid and transient increase in the [Ca^2+^]_cyt_ concentration is observed 40 sec after the application of *Vd* preparation (Figure 11A). Discharge at the end of the experiment demonstrates that less than 5% of the reconstituted aequorin was consumed after the stimuli, which ensures that the amount of aequorin in the sample is not limiting for the Ca^2+^ signal (data not shown). The [Ca^2+^]_cyt_ reached a peak of ~ 400 nM after 90 to 120 sec (Figure 11A). Subsequently the Ca^2+^ levels steadily decreased. No [Ca^2+^]_cyt_ elevation is observed with the PBS buffer treatment (Figure 11A). The magnitude of the [Ca^2+^]_cyt_ response is dose-dependent (data not shown). Furthermore, an Arabidopsis *cytoplasmic calcium elevation mutant1* (*cycam1*) which does not show [Ca^2+^]_cyt_ elevation in response to exudate preparation from various pathogenic fungi [45] also failed to induce [Ca^2+^]_cyt_ elevation in response to the *Vd* preparation (Figure 11B). This indicates that *cycam1* is impaired in the response to exudate preparations from various pathogens. Furthermore, we crossed the apoaeqorin gene into the *glr2.4*, *glr2.5* and *glr3.3* knock-out background. Figure 11B demonstrates that the *Vd* exudate preparation induced [Ca^2+^]_cyt_ elevation in the knock-out backgrounds, indicating that these putative plasma membrane-localized transporters do not participate in the Ca^2+^ uptake from the extracellular space, although the gene *GLR2.4* was upregulated in *Vd*-infected seedlings (Figure 5).

To investigate whether [Ca^2+^]_cyt_ elevation is required for disease development, *cycam1* was infected with *Vd* and the development of the mutant seedlings was compared to that of the WT seedlings. No obvious difference of the disease symptoms in the aerial parts could be detected, which suggests that [Ca^2+^]_cyt_ elevation is not essential for *Vd* propagation (Additional file 1: Figure S6).

## Discussion

Our data demonstrate that *Pi* is a very efficient biocontrol agent for *Vd* wilt in Arabidopsis. *Pi* restricts *Vd* growth both on agar plates (Figure 1) and in Arabidopsis roots, in particular when they were first colonized by *Pi* prior to infection with *Vd* (Figure 7). Molecular and biochemical analyses demonstrate that the reduced growth rate of *Vd* in *Pi*-pretreated Arabidopsis roots retards defense gene expression (Figure 5), the accumulation of defense-related phytohormones (Figure 6) and stomata closure (Figure 4). The performance of the seedlings is significantly better (Figure 2) and this also continues after shifting the seedlings to vermiculite for a longer period of time (Figure 9). *Pi* not only inhibits growth of *Vd* mycelia in Arabidopsis roots, but also prevents the spread of the pathogen to the aerial parts of the plant (Figure 7). Furthermore, microsclerotia formation is strongly reduced (Figure 8). Previously, several soil-borne bacteria have been identified as biocontrol agents for Verticillium wilt [29,46-48]. *Vd* can induce antimicrobial metabolites such as rutin in potato [49] or pathogenesis-related proteins in Arabidopsis [12] which participates in pathogen resistance. Prieto et al. [50] demonstrated that root hair colonization plays an important role in *Pseudomonas* spp.-mediated biocontrol activity against Verticillium wilt in olive roots. Furthermore, the *Bacillus subtilis* strain NCD-2 functions as a biocontrol agent against cotton Verticillium wilt, and the cotton PhoR/PhoP, two component regulatory systems, were involved in the biocontrol capability of the bacterium [51]. Also quorum sensing is involved in the biocontrol activity of *Serratia plymuthica* against *Vd* [52]. Moderate drought influences the effect of arbuscular mycorrhizal fungi as biocontrol agents against Verticillium-induced wilt in pepper [53]. It appears that quite different mechanisms control the fungal spread, probably because of the complicated lifestyle of the pathogen which allows microbial interference at different levels and in different plant tissues.

An increasing number of genes were recently identified to be involved in establishing partial resistance to Verticillium wilts (cf. Background). Pathogen attack including root colonization by *Vd* is associated with stomata closure as one of the first line of plant defense (Figure 4). *RabGAP22* is required for defense against *V. longisporum* and contributes to stomatal immunity [20]. *RabGAP11* gene is upregulated by *Vd* and repressed by *Pi* (Figure 5). Finally, defensins play a role in the plant defense against *Vd* [19].

Control of microsclerotia formation is crucial for preventing Verticillium spread in nature and agriculture. Our data demonstrate that *Pi* is quite efficient in restricting microsclerotia formation in Arabidopsis roots (Figure 8), presumable because the pathogen cannot grow fast enough in the presence of *Pi*. Microsclerotia formation is also suppressed by Verticillium itself, i.e. by the fungal transcription activator of adhesion Vta2, and fungi impaired in Vta2 are unable to colonize plants and induces disease symptoms [21]. Taken together, *Pi* restricts *Vd* growth as well as hyphal and microslerotia propagation, which - in turn - causes that the plant defense processes get activated at a lower level compared to *Vd* treatments which might depend on *Pi*-plant-*Vd* interaction-pattern and the attack strategy of *Vd*. This is not only important for better performance of individual plants, but has also severe long-term consequences for the control of the *Vd* spread *via* microsclerotia in ecosystems and agricultural areas.

GRL homologs are associated with Ca^2+^ influx through the plasma membrane. Figure 5 demonstrates that the mRNA level for *GLR2.4* is upregulated in the leaves of *Vd*-infected Arabidopsis seedlings and these responses are restricted by a pretreatment of the seedlings with *Pi*. GLR3.3 is involved in plant defense and resistance to *Hyaloperonospora arabidopsidis* [44]. The protein also mediates glutathione-triggered [Ca^2+^]_cyt_ transients, transcriptional changes, and innate immunity responses in Arabidopsis [54]. *GLR2.5* is upregulated in Arabidopsis cell cultures upon wounding [43] and *GLR2.4* is induced by nematodes in Arabidopsis roots [42]. GLR2.4, also called AUGMIN subunit 8, is a microtubule plus-end binding protein that promotes microtubule reorientation in hypocotyls [55,56]. Microtubules and microtubule orientation are important for plant defense and immunity [56,57] and also involved in *Vd*-Arabidopsis interaction. Hu et al. [18] demonstrated that histone H2B monoubiquitination is involved in regulating the dynamics of microtubules during the defense response to *Vd* toxins in Arabidopsis. Yuan et al. [58] showed that *Vd* toxins disrupted microfilaments and microtubules in Arabidopsis suspension-cultured cells. Figure 11A shows that exudate compounds from *Vd* induces [Ca^2+^]_cyt_ elevation in Arabidopsis roots. In order to test whether the [Ca^2+^]_cyt_ elevation is mediated by one of the three GLRs, we generated transgenic *glr3.3*, *glr2.5* and *glr2.4* knock-out lines in the apoaequorin background and found that the [Ca^2+^]_cyt_ response is not controlled by the three GLRs (Figure 11B), although the mRNA level of *GLR2.4* is upregulated upon *Vd* infection (Figure 5). This suggests that GLRs have different functions in the *Vd*-Arabidopsis interaction. However, an ethylmethansulfonate-induced Arabidopsis mutant named *cycam1* which is unable to induce [Ca^2+^]_cyt_ elevation in response to exudate preparations from *Alternaria brassicae*, *Rhizoctonia solani*, *Phytophthora parasitica* var. *nicotianae* and *Agrobacterium tumefaciens* [45] did not respond to the *Vd* exudate preparation (Figure 11B). This demonstrates that at least one of the *Vd*-induced signaling events leading the opening of Ca^2+^ channels or the channels themselves are identical to those responding to exudate preparations from other pathogens [45]. However, the reduced Ca^2+^ response in the *cycam1* mutant does not affect the disease development. It remains to be determined which is the active compound inducing the [Ca^2+^]_cyt_ response in Arabidopsis roots, and what is the mutated gene in the *cycam1* mutant.

Several exudated compounds have been postulated to induce pathogenicity in plants. Klosterman et al. [3] proposed that based on the sequence information of Verticillium species, pathogenicity may be caused by a cocktail of different compounds and elicitors with different functions in the complex pathogenicity procedure. A Verticillium crude toxin preparation has been often used, although the exact composition of this preparation and the role of the individual compounds are not clear. For instance, recently Yao et al. [59] have demonstrated that the *Vd* toxin preparation stimulates nitric oxide production in Arabidopsis which serves as a signaling intermediate downstream of H_2_O_2_ to modulate dynamic microtubule cytoskeleton. This may link the *Vd* toxin function again to GLR2.4, who’s mRNA level is upregulated after *Vd* infection (Figure 5). Wang et al. [60] reported on the purification and characterization of a novel hypersensitive-like response-inducible protein elicitor named PevD1 from *Vd* that induces resistance responses in tobacco. The relationship of the bioactive compound that induces the [Ca^2+^]_cyt_ response to the toxins which induce disease responses needs to be investigated.

Interestingly, we did not observe a linear relationship between the propagation of *Vd* in the seedlings and the accumulation of defense-related phytohormone levels. For instance, the phytohormone levels were always high when the seedlings were exposed to *Vd*, irrespective of whether they were exposed to *Vd* alone, pretreated with *Pi* or first with *Vd* followed by *Pi* (Figure 6), although, growth of *Vd* was strongly reduced by the *Pi* pretreatment (Figure 1). This suggests that even low infection rates of *Vd* are sufficient to stimulate the accumulation of the defense hormones. This might be a precaution, although propagation of *Vd* is inhibited when the roots were pretreated with *Pi*.

Various reports showed the involvement of plant hormones in the control of Verticillium growth in Arabidopsis. Stabilization of cytokinin levels enhances Arabidopsis resistance against *V. longisporum* [17]. The fungus also requires JA-dependent COI1 function in roots to elicit disease symptoms in Arabidopsis shoots [15]. Ethylene perception *via* the receptor ETR1 is required for *Vd* infection in Arabidopsis [11]. Enhanced resistance of *etr1-1* plants, but not of SA-, JA- or other ET-deficient mutants against *Vd* infection indicate a crucial role of ETR1 in defense against this pathogen. We observed a particularly striking resistance of the Arabidopsis *ein3* mutant against *Vd* infection *in vivo* and *in vitro* (Additional file 1: Figure S5). This is consistent with the reports by Pantelides et al. [11] for *etr1*, although they did not observe a significant role of EIN3 in their studies. Our data demonstrate that EIN3 plays an important role in pathogenicity and will provide an important tool to identify EIN3-regulated genes which are required for *Vd* disease development. Furthermore, the ET level in the *ein3* mutant exposed to *Vd* is much higher compared to *Vd*-exposed WT seedlings (Figure 10C). This suggests a feedback loop by which the lack of EIN3-induced defense responses in the *ein3* mutant results in an additional stimulation of ET synthesis.

## Conclusions

In summary, our data demonstrate that *Pi* is a very efficient biocontrol agent for *Vd*. This is mainly caused by the restriction of *Vd* growth in the presence of *Pi*. There appears to be additional mechanisms which prevent pathogenicity of *Vd* in the presence of *Pi*. For instance, the phytohormone levels accumulate to comparable levels in *Vd* and *1P2V* seedlings, although *Vd* propagation is restricted in the presence of *Pi* (Figure 1). Since *Pi* pretreatment severely reduces defense gene expression in spite of a comparable phytohormone level in these tissues, additional signals from *Pi* must participate in the regulation of the immune response against *Vd*.

## Methods

### Growth conditions of seedlings and fungi

*A. thaliana* wild-type (ecotype Columbia-0) seeds, seeds of the *glr2.4*, *glr2.5*, *glr3.3* and *ein3* mutants as well as of *cycam1* mutant [45] were surface-sterilized and placed on Petri dishes with MS media [61]. After cold treatment at 4°C for 48 h, plates were incubated for 11 days at 22°C under long day conditions (16 h light/8 h dark; 80 μmol m^−2^ sec^−1^). *Pi* was grown for 3-4 weeks on KM medium as described previously [62]. For detailed information see Section A and B in Johnson et al. [63]. *Vd* (FSU-343, Jena Microbial Resource Center, Germany) was grown for 2-3 weeks on Potato Dextrose Agar (PDA) medium [64].

### Co-cultivation assays

For co-cultivation assays 13 day-old *A. thaliana* seedlings of equal size were used. Co-cultivation of *A. thaliana* and the fungi *Pi* and/or *Vd* was performed under *in vitro* culture conditions on a nylon membrane on PNM media as described by Johnson et al. ([63], Section C1 - Method 1) with a few modifications. *Vd* was grown for 12 days and *Pi* for 10 days on the membrane on top of PNM medium in Petri dishes. 13-day old Arabidopsis seedlings were then transferred to the *Pi* or *Vd* plates, or mock-treated (no fungal mycelium; C). For the shifting experiments, the seedlings were transferred to plates with the other fungus after 4 days (from *Vd* to *Pi* or *vice-versa*). Including the (C), five different treatments were compared: (1) Arabidopsis seedlings grown without *Pi* or *Vd* (C); (2) without *Pi* and with *Vd* (*Vd*); (3) with *Pi* and without *Vd* (*Pi*); (4) with *Pi* for 4 days before transfer to *Vd* plates (*1P2V*) and (5) with *Vd* for 4 days before transfer to *Pi* plates (*1V2P*). The seedlings were harvested between 1 and 21 days after exposure to the first fungus (or mock-treatment) for further analysis. A time scheme is shown in Additional file 1: Figure S1. The light intensity (80 μmol m^−2^ s^−1^) was checked weekly. Shoots and roots were harvested separately for DNA and RNA analyses.

### Long term co-cultivation in sterile vermiculite

30 g vermiculite was placed into one Magenta box (Sigma-Aldrich, Germany) and autoclaved at 121°C for 30 min. After the addition of 40 ml of sterile liquid PNM medium, Arabidopsis seedlings grown in Petri dishes for 10 days were transferred to the sterile vermiculite boxes (1 plant per box). For each treatment, 16 seedlings were analyzed. After 10 days, the number of survived plants, their biomass and fungal DNA content were determined.

### Gene expression analysis

RNA was isolated from shoots and reverse-transcribed for Real-time quantitative PCR analysis, using an iCycler iQ Real-time PCR detection system and iCycler software version 2.2 (Bio-Rad). Total RNA was isolated from 5 independent biological experiments of Arabidopsis shoots. cDNA was synthesized using the Omniscript cDNA synthesis kit (QIAGEN) using 1 μg RNA. For the amplification of the RT-PCR products, iQ SYBR Green Supermix (Bio-Rad) was used according to the manufacturer’s protocol in a final volume of 20 μl. The iCycler was programmed to 95°C 3 min, 40 × (95°C 30 sec, 57°C 15 sec, 72°C 30 sec), 72°C 10 min, followed by a melting curve program 55°C to 95°C in increasing steps of 0.5°C. All reactions were performed in triplicate. The mRNA levels for each cDNA probe were normalized with respect to the glycerin-aldehyde-3-phosphate dehydrogenase (*GAPDH*) mRNA level. The primer pairs are given in Additional file 1: Table S1.

### Quantification of fungal DNAs by PCR

Genomic DNA extraction was conducted with DNeasy Plant Mini Kit. 12.5 ng DNA was taken for PCR template. The reactions were performed with gene-specific primers, as given in Additional file 1: Table S1. For details see Camehl et al. [65].

### Dual culture of *Pi* and *Vd*

Dual culture of *Pi* and *Vd* on agar plates was performed as described by Johnson et al. [66]. A *Pi* plug with 5 mm diameter was placed at one end of a PDA plate and a *Vd* plug of the same size at the other end of the plate. The plates were incubated at 22-24°C in dark and 75% relative humidity. Photos were taken after 3 weeks of co-cultivation.

### Percentage disease index (PDI) calculation

Disease index was calculated with the following formula:

$$ \mathrm{P}\mathrm{D}\mathrm{I}=\frac{{\mathrm{n}}_1\kern0.1em {\mathrm{x}}_1+{\mathrm{n}}_2\kern0.1em {\mathrm{x}}_2+{\mathrm{n}}_3\kern0.1em {\mathrm{x}}_3+{\mathrm{n}}_4\kern0.1em {\mathrm{x}}_4+{\mathrm{n}}_5\kern0.1em {\mathrm{x}}_5}{\mathrm{Total}\kern0.5em \mathrm{number}\kern0.5em \mathrm{of}\kern0.5em \mathrm{leaves}\kern0.5em \mathrm{observed}\times \mathrm{maximum}\kern0.5em \mathrm{grade}}\times 100 $$

n_1-5_ = number of affected leaves of the respective disease.

Severity grade (0-5), x_1-5_ = disease severity grade based on the percentage of affected leaf area. 1, 1% ≤ × ≤ 10%; 2, 10% < × ≤ 20%; 3, 20% < × ≤ 30%; 4, 30% <× ≤ 40%; 5, × > 40%; ×100: calculated in percentage scale. Disease severity was estimated on the basis of affected leaf area. 1-5 disease severity grades were described by Naik and Lakkund [67,68].

### Quantification of jasmonic acid (JA), JA-isoleucine (JA-Ile), abscisic acid (ABA), salicylic acid (SA), oxophytodinoic acid (OPDA) and ethylene (ET)

Independent samples of 250 mg shoot material were collected from each treatment. Phytohormone extractions (JA, JA-Ile, ABA, SA and OPDA) were performed by adding 1 ml ethyl-acetate containing 60 ng of D_2_-JA and 40 ng of D_6_-ABA, D_4_-SA and JA-^13^C_6_-Ile (OPDA has the same internal standerd as JA) to 100 mg ground tissues. All samples were then vortexed for 10 min and centrifuged at 13,000 rpm for 20 min at 4°C. The supernatants were collected and evaporated to dryness at 30°C using a vacuum concentrator. Residues were resuspended in 500 μl MeOH:H_2_O (70:30, v/v) and centrifuged at 13,000 rpm for 10 min. The supernatants were collected and measured with the API 3200 LC-MS/MS system (Applied Biosystems, Framingham, USA) as previously described [69].

For ET measurements, 100 mg shoot material from each treatment was collected into 4 ml vials (Roth, Germany). After 4 h ET accumulation, the measurement was performed with the ETD-300 ethylene detector (Sensor Sense B.V., Nijmegen, The Netherlands).

***Chlorophyll content*** was determined according to Yang et al. [70] and based on g fresh weight.

### Quantification of microsclerotia

Roots of Arabidopsis seedlings from the 3 treatments with *Vd* were harvested after 3 weeks of co-cultivation in Petri dishes and transferred to a microscopic glass slide with 80 μl lactic acid/glycerol/H_2_O (1:1:1). The number of the microsclerotia formed in the roots was calculated averagely per root visually under the light microscope (magnification: x200). The experiment was performed 3 times independently and for each treatment the roots of 12 seedlings were analysed.

### Cytoplasmic Ca^2+^ ([Ca^2+^]_cyt_) measurement

Aequorin based luminescence measurements were performed using 21-day old individual wild-type (WT) plants and mutants grown in Hoagland medium [71]. WT aequorin (pMAQ2) plants served as control [72]. Mutants (*glr2.4*, *glr2.5* and *glr3.3*) were crossed back to wild-type expressing aequorin. After 2 generation selection based on [Ca^2+^]_cyt_ responses and RT-PCR of T-DNA insertion examination, the homozygote seeds were used for the described experiments. Primers used for homozygosity tests are given in Additional file 1: Table S1. For [Ca^2+^]_cyt_ measurements, approximately 70% of the roots per seedling was dissected and incubated overnight in 150 μl of 7.5 μM coelentrazine (P.J.K. GmbH, Germany) in the dark at 20°C in a 96 well plate (Thermo Fischer Scientific, Finland, cat. no. 9502887). Bioluminescence counts from roots were recorded as relative light units (RLU) with a microplate luminometer (Luminoskan Ascent, version 2.4, Thermo Electro Corporation, Finland).

### Preparation of exudates from mycelia of *Vd*

A 5 mm *Vd* fungal plug was inoculated in Czapek’s medium as described in Zhen et al. [73] and grown for 3 weeks. Then, the fungal culture was filtered through double layers of filter paper and the filtrate was centrifuged at 10,000 *g* for 30 min to remove the spores. The supernatant was dialyzed with a dialysis membrane (MWCO) (Spectra/Por® Float-A-lyzer®) in 10 mM phosphate buffer pH 7.0 at 4°C for 24 h. The dialyzed solution was frozen and lyophilized. The powder was dissolved in distilled water and the solution was filtered through a 0.45 μm pore size Millipore filter (Roth, Germany). The resulting filtrate was used as exudate for further experiments.

### Statistics

All statistical analyses were performed using Excel or SPSS 17.0 (SPSS Inc., Chicago, IL, USA) for ANOVA.

### Availability of supporting data

All the supporting data are included as additional file.

## Additional file

Additional file 1: Figure S1. Co-cultivation time scheme. The seeds were first kept at 4°C in the dark for 2 days and were then transferred to a light/dark cycle at 22°C for 9 days. These seedlings were used for the experiments, by either transferring them to a plate with *Vd* or *Pi* (or no fungus, control, C) at day 0. The seedlings were harvested 10, 14 or 21 days later. In case of transfer from *Vd* to *Pi* or *vice versa*, the transfer occurred at day 4. **Figure S2.** Induction of *GLR* genes in shoots of Arabidopsis seedlings after 1 and 14 days. **Figure S3.** Phenotype of *ein3-1* and WT after 21 days of co-cultivation following the 5 treatments described in Methods. **Figure S4.** ET content in shoots of *ein3-1* seedlings after 3 weeks. **Figure S5.** Phenotypes of WT and *ein3-1* after *Vd* spore inoculation *in vivo* and *in vitro*. **Figure S6.** Phenotype of WT and *cycam1* mutant 21 days after *Vd* inoculation. **Table S1.** Primer list for RT-PCR and PCR analysis.

## Abbreviations

*Vd*: *Verticillium dahliae*
*Pi*: *Piriformospora indica*
[Ca^2+^]_cyt_: Cytosolic calcium
*cycam1*: *Cytosolic calcium elevation mutant1*
*glr*: *Glutamate receptor* mutants
*ein3*: *Ethylene-insensitive3 mutant*
JA: Jasmonic acid
JA-Ile: Jasmonyl-isoleucine
ABA: Abscisic acid
SA: Salicylic acid
OPDA: Oxophytodinoic acid
ET: Ethylene
WT: Wild-type
