# Supplementary material for: The beneficial fungus Piriformospora indica protects Arabidopsis from Verticillium dahliae infection by downregulation plant defense responses
Source: BMC Plant Biol. 2014 Oct 9;14:268. doi: 10.1186/s12870-014-0268-5 (PMC4198706; doi:10.1186/s12870-014-0268-5)
Supplement: Additional file 1: Figure S1. — Co-cultivation time scheme. The seeds were first kept at 4°C in the dark for 2 days and were then transferred to a light/dark cycle at 22°C for 9 days. These seedlings were used for the experiments, by either transferring them to a plate with Vd or Pi (or no fungus, control, C) at day 0. The seedlings were harvested 10, 14 or 21 days later. In case of transfer from Vd to Pi or vice versa, the transfer occurred at day 4. Figure S2. Induction of GLR genes in shoots of Arabidopsis seedlings after 1 and 14 days. Figure S3. Phenotype of ein3-1 and WT after 21 days of co-cultivation following the 5 treatments described in Methods. Figure S4. ET content in shoots of ein3-1 seedlings after 3 weeks. Figure S5. Phenotypes of WT and ein3-1 after Vd spore inoculation in vivo and in vitro. Figure S6. Phenotype of WT and cycam1 mutant 21 days after Vd inoculation. Table S1. Primer list for RT-PCR and PCR analysis. [file 12870_2014_268_MOESM1_ESM.pdf]

The beneficial fungus *Piriformospora indica* protects Arabidopsis from *Verticillium dahliae* infection by downregulation plant defense responses

Chao Sun[1], Yongqi Shao[2], Khabat Vahabi[1], Jing Lu[2,6], Samik Bhattacharya[2], Sheqin Dong[3], Kai-Wun Yeh[4], Irena Sherameti[1], Binggan Lou[5], Ian T. Baldwin[2], Ralf Oelmüller[1]*

[1]Institute of Plant Physiology, Friedrich-Schiller-University Jena, Dornburger Str. 159, 07743 Jena, Germany

[2]Max Planck Institute for Chemical Ecology, Hans-Knöll-Straße 8, D-07745 Jena, Germany

[3]College of Life Sciences, Yangtze University, Jingzhou, China

[4]Institute of Plant Biology, National Taiwan University, Taipei, Taiwan

[5]Institute of Biotechnology, Zhejiang University, Hangzhou 310058, China

[6]Institute of Insect Sciences, Zhejiang University, Hangzhou 310058, China

*Author for correspondence
Tel: +49 3641 949231
Fax: +49 3641 949232
e-mail: b7oera@uni-jena.de

Supplementary Figures

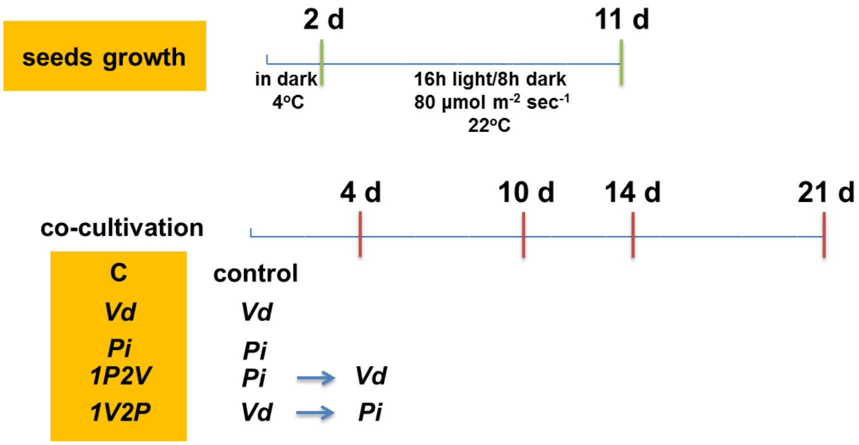

Figure S1. Co-cultivation time scheme. The seeds were first kept at 4°C in the dark for 2 days and were then transferred to a light/dark cycle at 22°C for 9 days. These seedlings were used for the experiments, by either transferring them to a plate with *Vd* or *Pi* (or no fungus, control, C) at day 0. The seedlings were harvested 10, 14 or 21 days later. In case of transfer from *Vd* to *Pi* or *vice versa*, the transfer occurred at day 4.

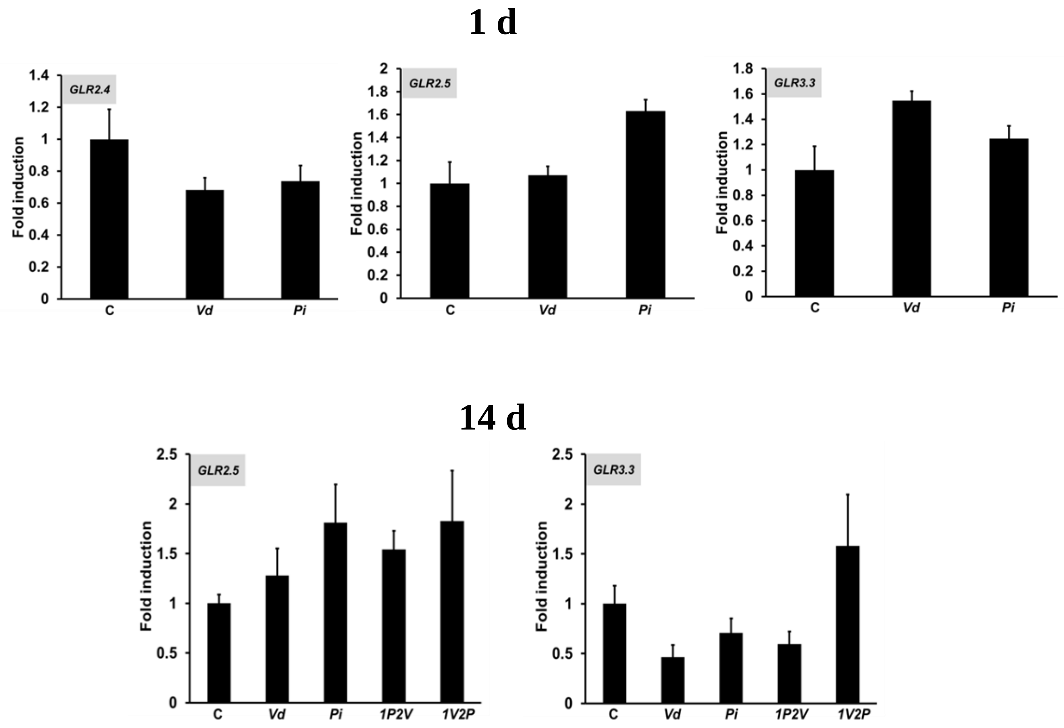

Figure S2. Induction of *GLR* genes in shoots of Arabidopsis seedlings after 1 and 14 days.

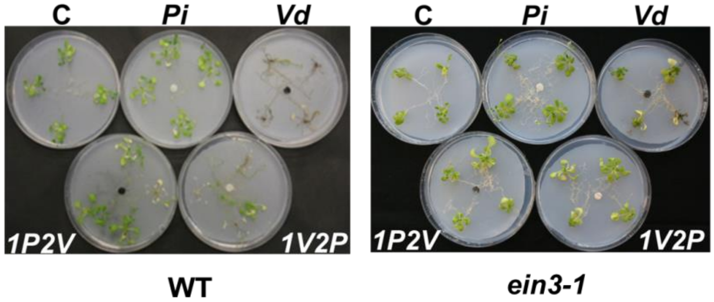

Figure S3. Phenotype of *ein3-1* and WT after 21 days of co-cultivation following the 5 treatments described in Methods and material.

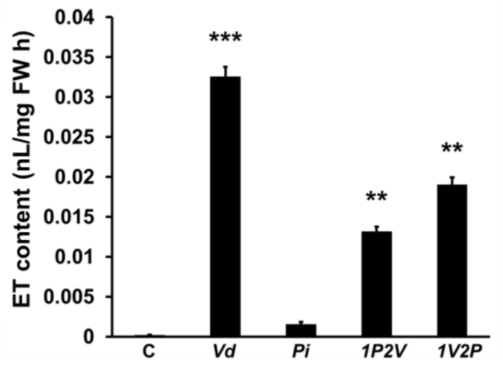

Figure S4. ET content in shoots of *ein3-1* seedlings after 3 weeks.

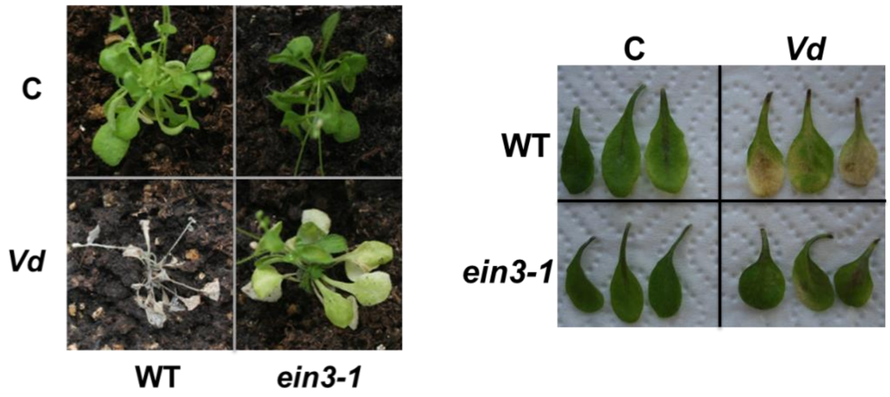

Figure S5. Phenotypes of WT and *ein3-1* after *Vd* spore inoculation *in vivo* and *in vitro*.

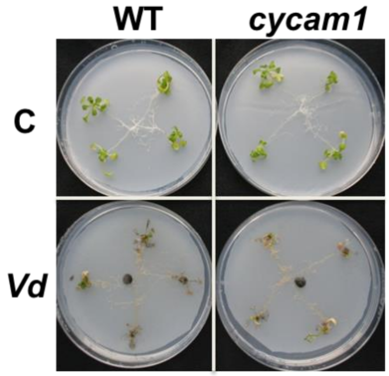

Figure S6. Phenotype of WT and *cycam1* mutant 21 days after *Vd* inoculation.

Table S1. Primer pairs used for real-time PCR and PCR analysis.

PR1	At2g14610	5´-TGTATGAGTCTGCAGTTGCC-3´<br>5´-CAACTGCAGACTCATACA -3´
PDF1.2	At5g44420	5´-CTTGTGTGCTGGGAAGACATA-3´<br>5´-AGCACAGAAGTTGTGCGAGAA-3´
PR-2	At3g57260	5´-TCTTCTCAGCCTTGTAATAGC-3´<br>5´-TGTTTGTAAAGAGCCACAACG-3´
ERF1	At3g23240	5´-CCTTCCGATCAAATCCGTAAG-3´<br>5´-TCCCGAGCCAAACCCTAATAC-3´
VSP2	At5g24770	5´-GAGCTGACTACGTTGTTGAG-3´<br>5´-GGAGACAATGTCAAGGTCGG-3´
GLR2.4 (for Salk line)	At4g31710	5´-CTTCTCGTCCAGAATCTCGGACC-3´<br>5´-GTCAAGGAAAGGGCTCGTTGCAG-3´
GLR2.5 (for Salk line)	At5g11210	5´-CCATGTGTCTCACTGTTGGTCG-3´<br>5´-GCAGATGAGGAGTTCAGGGAACAG-3´
GLR3.3 (for Salk line)	At1g42540	5´-ACGGCTGCAGTGAATCTTTT-3´<br>5´-AGAAAGCAACAACCCGTGAC-3´
RabGAP11		5´-GCACCTCCCACTGATGATTT-3´<br>5´-AACTGCCCTGCCATACTTTG-3´
GLR2.4 (for RT-PCR)	At4g31710	5'-AGGGAAAACATGTGATTGTGC-3'<br>5'-TCCAATAATGCCCTTGTCAAG-3'
GLR2.5 (for RT-PCR)	At5g11210	5'-CCTTTTAGGTGTTCAAAGGGG-3'<br>5'-CAGCAGAAGAGAGGTACACCG-3'
GLR3.3 (for RT-PCR)	At1g42540	5'-GATGCTGCATATGGTTGTGTG-3'<br>5'-GTTGAACGATAAGCTTGCGAG-3'
P. indica	elongation factor	5'-CGCAGAATACAAGGAGGCC-3'<br>5'-CGTATCGTAGCTCGCCTGC-3'
V. dahliae		5'-CACATTCAGTTCAGGAGACGGA-3'<br>5'- CCGAAATACTCCAGTAGAAGG-3'
GapDH	At3g04120	5'-GAGCTGACTACGTTGTTGAG-3'<br>5'-GGAGACAATGTCAAGGTCGG-3'
